# Left atrial dysfunction in bicuspid aortic valve patients with severe aortic stenosis is associated with post-operative atrial fibrillation following aortic valve replacement

**DOI:** 10.1093/ehjopen/oeae020

**Published:** 2024-03-28

**Authors:** Johan O Wedin, Sergey Rodin, Frank A Flachskampf, Oscar E Simonson, Johan Pallin, Jonathan Hörsne Malmborg, Stefan K James, Elisabeth Ståhle, Karl-Henrik Grinnemo

**Affiliations:** Department of Surgical Sciences, Uppsala University Hospital, entrance 70, 1st floor, SE-751 85 Uppsala, Sweden; Department of Cardiothoracic Surgery, Uppsala University Hospital, entrance 50, 4th floor, SE-751 85 Uppsala, Sweden; Department of Surgical Sciences, Uppsala University Hospital, entrance 70, 1st floor, SE-751 85 Uppsala, Sweden; Department of Cardiothoracic Surgery, Uppsala University Hospital, entrance 50, 4th floor, SE-751 85 Uppsala, Sweden; Department of Medical Sciences, Uppsala University Hospital, entrance 40, 5th floor, SE-751 85 Uppsala, Sweden; Department of Clinical Physiology, Uppsala University Hospital, entrance 40, 5th floor, SE-751 85 Uppsala, Sweden; Department of Surgical Sciences, Uppsala University Hospital, entrance 70, 1st floor, SE-751 85 Uppsala, Sweden; Department of Cardiothoracic Surgery, Uppsala University Hospital, entrance 50, 4th floor, SE-751 85 Uppsala, Sweden; Department of Cardiothoracic Surgery, Uppsala University Hospital, entrance 50, 4th floor, SE-751 85 Uppsala, Sweden; Department of Cardiothoracic Surgery, Uppsala University Hospital, entrance 50, 4th floor, SE-751 85 Uppsala, Sweden; Department of Medical Sciences, Uppsala University Hospital, entrance 40, 5th floor, SE-751 85 Uppsala, Sweden; Department of Cardiology, Uppsala University Hospital, entrance 40, 5th floor, SE-751 85 Uppsala, Sweden; Uppsala Clinical Research Center, Uppsala Science Park, Hubben, Dag Hammarskjölds väg 38, SE-751 85 Uppsala, Sweden; Department of Surgical Sciences, Uppsala University Hospital, entrance 70, 1st floor, SE-751 85 Uppsala, Sweden; Department of Cardiothoracic Surgery, Uppsala University Hospital, entrance 50, 4th floor, SE-751 85 Uppsala, Sweden; Department of Surgical Sciences, Uppsala University Hospital, entrance 70, 1st floor, SE-751 85 Uppsala, Sweden; Department of Cardiothoracic Surgery, Uppsala University Hospital, entrance 50, 4th floor, SE-751 85 Uppsala, Sweden

**Keywords:** Aortic stenosis, Bicuspid aortic valve, Left atrial reservoir strain

## Abstract

**Aims:**

To investigate (i) the association between pre-operative left atrial (LA) reservoir strain and post-operative atrial fibrillation (AF) and (ii) the incidence of post-operative ischaemic stroke events separately in bicuspid aortic valve (BAV) and tricuspid aortic valve (TAV) patients after surgical aortic valve replacement for isolated severe aortic stenosis (AS).

**Methods and results:**

We prospectively enrolled 227 patients (*n* = 133 BAV and *n* = 94 TAV) with isolated severe AS scheduled for aortic valve replacement. A comprehensive intra- and inter-observer validated pre-operative echocardiogram with an analysis of LA reservoir strain was performed. Post-operative AF was defined as a sustained (>30 s) episode of AF or atrial flutter. The timing of neurological events was defined in accordance with the Valve Academic Research Consortium-3 criteria for stroke. Post-operative AF occurred in 114 of 227 patients (50.2%), with no difference between BAV and TAV patients (48.1 vs. 53.1%, *P* = 0.452). Persisting post-operative AF at discharge was more frequent in BAV patients (29.7 vs. 8.0%, *P* = 0.005). Pre-operative LA reservoir strain was independently associated with post-operative AF (odds ratio = 1.064, 95% confidence interval 1.032–1.095, *P* < 0.001), with a significant interaction between LA reservoir strain and aortic valve morphology (*P*_interaction_ = 0.002). The cumulative transient ischemic attack (TIA)/stroke incidence during follow-up was significantly higher in BAV patients (19.1 vs. 5.8% at 5 years).

**Conclusion:**

Pre-operative LA function was associated with post-operative AF after aortic valve replacement in BAV AS patients, while post-operative AF in TAV AS patients likely depends on transient post-operative alterations and traditional cardiovascular risk factors. TIA/stroke during follow-up was more common in BAV AS patients.

## Graphical Abstract

**Graphical Abstract:**
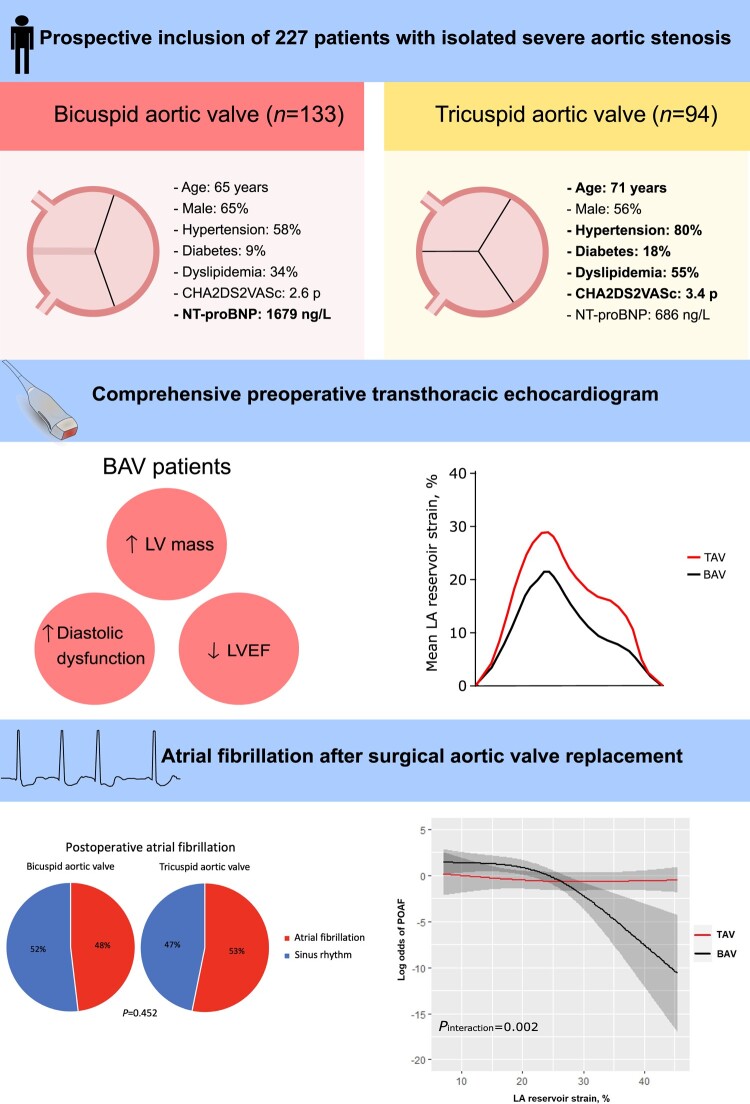

## Introduction

Post-operative atrial fibrillation (POAF), defined as new-onset atrial fibrillation (AF) occurring in the immediate period after surgery in patients with pre-operative sinus rhythm, is frequently observed after surgical aortic valve replacement (SAVR).^1^ The incidence remains high despite advancements in prophylactic strategies.^2–5^ Although often considered transient and self-terminating, it is a highly relevant complication associated with longer stay at the intensive care unit and total hospital stay, increased morbidity from heart failure and stroke, and increased mortality.^6–8^ The underlying mechanisms behind POAF are multifactorial and complex, with increasing age being the most important risk factor.^1,4,9^

Left atrial (LA) structural and functional remodelling is also an important predisposing risk factor for the development and maintenance of POAF.^10,11^ Left atrial function can be quantified using a two-dimensional speckle-tracking echocardiography–derived strain analysis.^12^ All three phases of the LA cardiac cycle (reservoir, conduit, and booster) can be quantified using a strain analysis, of which the LA reservoir strain has been validated against invasive left ventricular (LV) filling pressures.^13^ Impaired LA reservoir strain is associated with POAF after SAVR, independent of LA enlargement.^11,14,15^ It is also associated with new-onset AF in heart failure patients^16^ as well as in the general population.^17^ Impaired LA reservoir strain is also a risk factor for ischaemic stroke in patients with permanent AF.^18^

Tricuspid aortic valve (TAV) and congenital bicuspid aortic valve (BAV) degeneration represent the dominating aetiologies of severe aortic stenosis (AS) in the Western world. Despite the relatively low prevalence of BAV in the general population, it is one of the leading causes of AS in patients referred for SAVR. Tricuspid aortic valve predominates in older AS patients, while BAV is found in younger AS patients.^19^ Tricuspid aortic valve patients have more prevalent comorbidities compared with BAV patients.^20^ On the other hand, BAV patients with isolated severe AS demonstrate worse LV systolic and diastolic function, as well as LA remodelling, compared with TAV patients,^21^ factors that are closely related to the impairment of LA function.^22^

The incidence of POAF and TIA/stroke after SAVR for isolated AS in BAV and TAV patients and the association with pre-operative LA reservoir strain have not been investigated previously. This is of interest since we have recently demonstrated that BAV and TAV patients eligible for SAVR have completely different risk profiles, where surprisingly, BAV patients have increased LA volumes as well as significantly reduced LV ejection fraction (LVEF) at the time of surgery.^21^ This prospective study adds further support to the differentiated management of BAV and TAV patients with AS in the surgical guidelines.

## Methods

### Study design and patient selection

In this longitudinal prospective single-centre study, consecutive patients with isolated severe AS referred for SAVR to the Department of Cardiothoracic Surgery, Uppsala University Hospital, Uppsala, Sweden, between 1 January 2014 and 31 May 2021 were eligible for inclusion. Patients with pre-operative paroxysmal or permanent AF or atrial flutter were not considered for inclusion in the study cohort. Other exclusion criteria were history of coronary artery disease (prior percutaneous coronary intervention/coronary artery bypass grafting or significant coronary stenosis found on pre-operative coronary angiography), previous open-heart surgery, combined moderate-to-severe valvular stenosis or regurgitation, mitral annular calcification causing moderate or severe mitral stenosis, and concomitant surgical procedures other than ascending aortic surgery. Patients with insufficient echocardiogram quality to allow for LA strain analysis were excluded (*n* = 19 BAV and *n* = 25 TAV). The aortic valve morphology (BAV or TAV) was determined by the surgeon’s intra-operative description and documented immediately in the operating room. The study complied with the Declaration of Helsinki and was approved by the Regional Ethics Review Board.

### Echocardiographic imaging

The pre-operative transthoracic echocardiogram was analysed by a single observer in an offline software, and a deformation analysis was performed in the semi-automatic 2D Cardiac Performance Analysis platform (TomTec Imaging Systems GmbH, Unterschleißheim, Germany). Assessments of LV dimensions and systolic LV function, global longitudinal strain (GLS), and diastolic function were performed according to recommendations^23–25^ as described in the Supplementary material.

#### Left atrial reservoir strain

Left atrial reservoir strain was determined according to the proposed recommendations.^26^ In a non-foreshortened apical four-chamber view, the LA myocardial wall was semi-automatically traced. A region of interest was delineated after marking the septal and lateral edges of the mitral annulus and the centre of the LA roof, excluding the pulmonary veins and the LA appendage, dividing the LA wall into six segments (two inter-atrial septal segments, two atrial roof segments, and two lateral segments). Manual corrections were made if necessary. The end-diastole (QRS onset) was used as a zero reference point, and the LA reservoir strain was automatically calculated.

#### Reproducibility

Inter- and intra-observer variabilities for LA reservoir strain were analysed in a random sample of 20 patients. The analysis showed an excellent consistency of repeated measures (Pearson *r* and intra-class correlation coefficient >0.90, Supplementary material online, *Table S1* and *Figure S1A–D*). Reproducibility of conventional echocardiographic parameters has been reported previously.^21^

### Post-operative atrial fibrillation detection

All patients were continuously monitored on telemetry from the day of SAVR until hospital discharge. A 12-lead electrocardiogram was taken when necessary and routinely recorded in all patients on the third post-operative day. Post-operative atrial fibrillation was defined as a sustained (>30 s) episode of AF or atrial flutter.

### Outcome

The outcomes of interest were POAF and TIA or stroke during follow-up. The timing of neurological events was defined in accordance with the Valve Academic Research Consortium-3 criteria for stroke.^27^ Peri-procedural events were defined as occurring ≤30 days after the index procedure, where acute events occurred ≤24 h after the index procedure and sub-acute events occurred >24 h and ≤30 days after the index procedure. Early events were defined as occurring >30 days and ≤1 year after the index procedure, while late events were defined as occurring >1 year after the index procedure. Information on TIA/stroke events was obtained from electronic health records. The patients were followed up from the date of surgery until they experienced a TIA/stroke event, until their death, or until they were administratively censored (December 31 2021). We did not consider repeat events in the analysis.

### Statistical analysis

Continuous data are expressed as mean ± standard deviation (±SD), and categorical variables are expressed as numbers (%). Differences between BAV and TAV patients and between POAF subgroups were analysed using the independent samples *t*-test and the *χ*^2^ test or Fisher’s exact test (as appropriate) for continuous and categorical data, respectively. A receiver operating characteristic analysis was conducted to assess the overall performance of LA reservoir strain to predict POAF. The optimal discriminatory cut-off for LA reservoir strain was determined from the Youden index. This cut-off was used when dichotomizing LA reservoir strain for investigation of the incremental value of LA reservoir strain over standard echocardiographic parameters and the association with persisting POAF at discharge. Multivariable logistic regression analysis was used to assess an association between relevant parameters and POAF. The results are presented as odds ratio with 95% confidence intervals (CIs). The selection of covariates of interest was based on prior knowledge, and necessary adjustments were performed after the construction of directed acyclic graphs (see Supplementary material online, *Figure S2*). To investigate interaction effects between aortic valve morphology (BAV or TAV) and the selected parameters on POAF probability, a multiplicative interaction term was introduced in the multivariable logistic regression model if a significant main effect was observed. Post-operative TIA/stroke incidence for BAV and TAV patients was displayed using cumulative incidence function curves, accounting for death as a competing risk. We investigated the association between TIA/stroke as an outcome variable and LA reservoir strain and LA volume index as independent variables in an univariable Cox proportional hazards regression analysis. The results are presented as hazard ratio (HR) with 95% CI. R (CRAN project, version 4.0.2) was used for all statistical analyses. Cumulative incidence function curves were constructed in R (CRAN project, version 4.0.2), and competing risk analysis was conducted in the same software. A two-tailed *P-*value of <0.05 was considered statistically significant.

## Results

We included 227 patients in this prospective study. The mean follow-up was 1342 days for BAV patients and 1562 days for TAV patients (*P* = 0.029). Baseline characteristics of the BAV and TAV patients are summarized in *Table 1*. Bicuspid aortic valve patients were younger (65.3 vs. 71.2 years, *P* < 0.001) and had less prevalent hypertension (57.9 vs. 79.8%, *P* < 0.001), diabetes mellitus (9.0 vs. 18.1%, *P* = 0.044), hyperlipidaemia (33.8 vs. 55.3%, *P* < 0.001), lower creatinine levels (78 vs. 83 µg/L, *P* = 0.034), and higher N-terminal pro-brain natriuretic peptide levels (1679 vs. 686 ng/L, *P* = 0.015) compared with TAV patients.

**Table 1:** Pre-operative baseline characteristics of 227 patients with isolated severe aortic stenosis

	Bicuspid aortic valve (*N* = 133)	Tricuspid aortic valve (*N* = 94)	*P*-value
Demographic data			
Age, years	65.3 (±9.2)	71.2 (±7.1)	<0.001
Male gender, *n* (%)	87 (65.4)	53 (56.4)	0.168
Body mass index, kg/m^2^	27.1 (±4.5)	28.3 (±4.0)	0.032
Body surface area, m^2^	1.94 (±0.21)	1.93 (±0.21)	0.780
Physiological data			
Systolic blood pressure, mmHg	134 (±18.0)	140 (±13.4)	0.008
Diastolic blood pressure, mmHg	76 (±12.5)	77 (±9.0)	0.505
Resting heart rate, b.p.m.	74 (±16.8)	69 (±10.0)	0.002
Clinical data			
Diabetes mellitus, *n* (%)	12 (9.0)	17 (18.1)	0.044
Arterial hypertension, *n* (%)	77 (57.9)	75 (79.8)	<0.001
Hyperlipidaemia, *n* (%)	45 (33.8)	52 (55.3)	<0.001
Prior TIA/stroke	10 (7.5)	8 (8.5)	0.785
Chronic obstructive pulmonary disease, *n* (%)	8 (6.0)	10 (10.6)	0.204
Peripheral artery disease, *n* (%)	10 (7.5)	6 (6.4)	0.742
Smoking (previous or current), *n* (%)	52 (39.1)	38 (40.4)	0.840
CHA_2_DS_2_VASc	2.58 (±1.48)	3.40 (±1.37)	<0.001
Anti-hypertensive medication			
ACEi/ARB, *n* (%)	64 (48.1)	53 (56.4)	0.249
β-blocker, *n* (%)	46 (34.6)	50 (53.2)	0.004
CCB, *n* (%)	19 (14.3)	28 (29.8)	0.004
Diuretics, *n* (%)	28 (21.1)	28 (29.8)	0.121
Pre-operative levosimendan, *n* (%)	12 (9.0)	0 (0.0)	0.003
Laboratory data			
Haemoglobin, g/L	139 (±12.6)	139 (±12.7)	0.642
NT-proBNP, ng/L	1679 (±3575)	686 (±1130)	0.015
Creatinine, µg/L	78 (±16)	83 (±18)	0.034

ACEi, angiotensin-converting enzyme inhibitor; β-blocker, beta receptor blocker; ARB, angiotensin receptor II blocker; CCB, calcium channel blocker; NT-proBNP, N-terminal pro-brain natriuretic peptide.

Pre-operative echocardiographic characteristics of the BAV and TAV patients are summarised in *Table 2*. Bicuspid aortic valve patients had a significantly higher left ventricular mass index (LVMi) (134.3 vs. 103.5 g/m^2^, *P* < 0.001), worse pre-operative LVEF (55 vs. 60%, *P* < 0.001), and more prevalent diastolic dysfunction (70.9 vs. 44.3%, *P* < 0.001) compared with TAV patients. Indexed LA volume was higher (41 vs. 36 mL/m^2^, *P* < 0.001) and LA reservoir strain was lower in the BAV cohort (22.2 vs. 28.1%, *P* < 0.001).

**Table 2:** Pre-operative echocardiographic characteristics of 227 patients with isolated severe aortic stenosis

	Bicuspid aortic valve (*N* = 133)	Tricuspid aortic valve (*N* = 94)	*P*-value
LV size and systolic parameters			
IVS diastolic thickness, mm	12.8 (±1.8)	11.8 (±1.8)	<0.001
LVPW diastolic thickness, mm	12.0 (±1.7)	10.7 (±1.6)	<0.001
LV end-diastolic diameter, mm	51.4 (±6.9)	48.2 (±5.6)	<0.001
LV mass index, g/m^2^	134.3 (±37.3)	103.5 (±24.7)	<0.001
Relative wall thickness	0.47 (±0.09)	0.45 (±0.08)	0.073
LVEDV index, mL/m^2^	77.0 (±38.8)	63.6 (±14.2)	0.002
LVESV index, mL/m^2^	35.8 (±21.0)	26.1 (±9.3)	<0.001
LVEF, %	55 (±11)	60 (±7)	<0.001
GLS, % (*n* = 190)	−14.3 (±4.0)	−18.0 (±5.2)	<0.001
Aortic valve parameters			
AV maximal velocity, m/s	4.64 (±0.58)	4.59 (±0.52)	0.501
AV maximal gradient, mmHg	87.6 (±22.7)	85.7 (±20.0)	0.522
AV mean gradient, mmHg	55.2 (±15.7)	53.3 (±12.2)	0.329
AVA index, cm^2^/m^2^	0.40 (±0.12)	0.45 (±0.10)	0.002
Pressure recovery, mmHg	2.15 (±0.76)	2.59 (±0.75)	<0.001
LV diastolic parameters			
Left atrial volume index, mL/m^2^	41 (±10)	36 (±9)	<0.001
E wave, cm/s	0.83 (±0.23)	0.83 (±0.21)	0.786
A wave, cm/s	0.80 (±0.26)	1.00 (±0.23)	<0.001
E/A ratio	1.22 (±0.91)	0.88 (±0.35)	<0.001
Septal e′, cm/s	5.5 (±1.5)	5.8 (±1.5)	0.154
Lateral e′, cm/s	6.9 (±1.9)	6.8 (±2.0)	0.781
E/e′ septal	16.4 (±5.9)	15.1 (±4.9)	0.108
E/e′ lateral	13.1 (±5.0)	12.7 (±4.3)	0.605
TR Vmax, m/s	2.71 (±0.44)	2.50 (±0.31)	0.005
Diastolic dysfunction, *n* (%)	83 (70.9)	35 (44.3)	<0.001
LA reservoir strain, %	22.2 (±10.8)	28.1 (±9.4)	<0.001

LV, left ventricle; LVEF, LV ejection fraction; GLS, global longitudinal strain; LVEDV, LV end-diastolic volume; LVESV, LV end-systolic volume; IVS, interventricular septum; LVPW, LV posterior wall; AV Vmax, aortic valve maximal velocity; AVA, aortic valve area; TR Vmax, tricuspid regurgitation maximal velocity; LA, left atrium.

### Peri- and post-operative characteristics

The peri- and post-operative characteristics are summarised in *Table 3*. Bicuspid aortic valve patients required more post-operative inotropic support compared with TAV patients (28.6 vs. 12.8%, *P* = 0.005). The need for post-operative nitroglycerin infusion to lower blood pressure was higher for TAV patients (28.7 vs. 14.3%, *P* = 0.008). Peak C-reactive protein (CRP) levels (206 vs. 192 mg/L, *P* = 0.102) and peak creatine kinase muscle brain (CK-MB) levels (29 vs. 23 µg/L, *P* = 0.415) were similar for BAV and TAV patients. Maximal post-operative body weight increase, a marker of fluid balance, was similar for BAV and TAV patients (+3.64 vs. +3.47 kg, *P* = 0.591). The occurrence of significant pericardial effusion (9.0 vs. 7.5%, *P* = 0.673) and significant pneumothorax (3.8 vs. 2.1%, *P* = 0.702) did not differ between the two groups of patients.

**Table 3:** Post-operative characteristics of the whole cohort

	Bicuspid aortic valve (*N* = 133)	Tricuspid aortic valve (*N* = 94)	*P*-value
Post-operative atrial fibrillation, *n* (%)	64 (48.1)	50 (53.1)	0.452
ICU, days	2.20 (2.51)	1.68 (1.31)	0.073
Mechanical prosthetic valve, *n* (%)	36 (27.1)	6 (6.4)	<0.001
Weight increase from baseline, kg	3.64 (2.09)	3.47 (1.95)	0.591
Significant pericardial effusion, *n* (%)	12 (9.0)	7 (7.5)	0.673
Significant pneumothorax, *n* (%)	5 (3.8)	2 (2.1)	0.702
Peak C-reactive protein level, mg/L	206 (68)	192 (56)	0.102
Peak CK-MB level, µg/L	29 (68)	23 (22)	0.415
Inotropic support, *n* (%)	38 (28.6)	12 (12.8)	0.005
Nitroglycerin infusion, *n* (%)	19 (14.3)	27 (28.7)	0.008

ICU, intensive care unit; CK-MB, creatine kinase muscle brain.

### Post-operative atrial fibrillation

Post-operative atrial fibrillation occurred in 114 of 227 patients (50.2%), with no difference between BAV and TAV patients (48.1 vs. 53.1%, *P* = 0.452). The time to POAF onset was similar for BAV and TAV patients (2.72 vs. 2.49 days, *P* = 0.441). About 9% of BAV patients and 20% of TAV patients converted to sinus rhythm spontaneously (*P* = 0.095). Post-operative atrial fibrillation was managed similarly in BAV and TAV patients, with similar rates of treatment with amiodarone (75.0 vs. 82.0%, *P* = 0.264) and/or electrical cardioversion (48.4 vs. 34.0%, *P* = 0.143). Despite this, successful conversion to sinus rhythm was less often successful for BAV patients (POAF at discharge: 29.7 vs. 8.0%, *P* = 0.005).

The characteristics of BAV and TAV patients with POAF, displayed in Supplementary material online, *Table S2*, showed similar differences to those observed between overall BAV and TAV cohorts. In summary, BAV patients were younger and had a worse pre-operative systolic and diastolic heart function, including LA reservoir strain. Tricuspid aortic valve patients had significantly higher CHA_2_DS_2_VASc scores, reflecting significantly more prevalent cardiovascular comorbidities. Supplementary material online, *Table S3* summarises the difference between patients with and without POAF with regard to aortic valve morphology.

**Figure 1:**
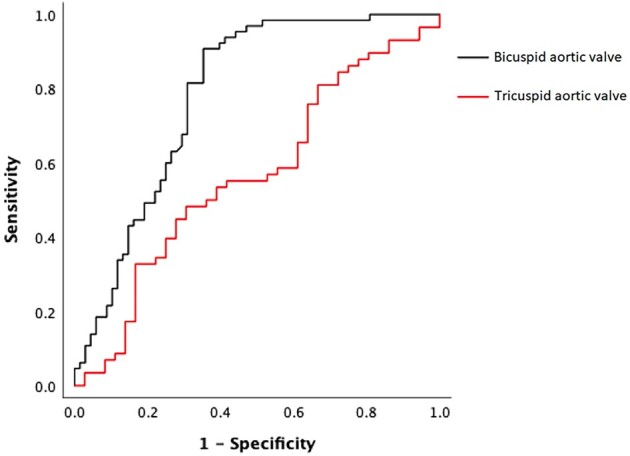
A receiver operating characteristics analysis showed that the diagnostic accuracy of left atrial reservoir strain to predict post-operative atrial fibrillation was different for bicuspid aortic valve and tricuspid aortic valve patients. The area under the curve for bicuspid aortic valve patients was 0.79 (95% confidence interval 0.70–0.86), and the area under the curve was 0.57 (95% confidence interval 0.44–0.67) for tricuspid aortic valve patients.

The rate of accuracy to predict POAF in the whole cohort with the use of LA reservoir strain was modest [area under the curve (AUC) = 0.672], with an optimal cut-off at 24.4% with a sensitivity of 70% and a specificity of 66%. The diagnostic accuracy for LA reservoir strain to predict POAF was different for BAV and TAV patients (*Figure 1*). The AUC for BAV patients was 0.79 (95% CI 0.70–0.86), with the cut-off value <24.4% with a sensitivity and a specificity of 91 and 65%, respectively. The corresponding values for TAV patients were the following: AUC = 0.57 (95% CI 0.44–0.67), cut-off 25.2% with a sensitivity of 48% and a specificity of 59%.

**Figure 2:**
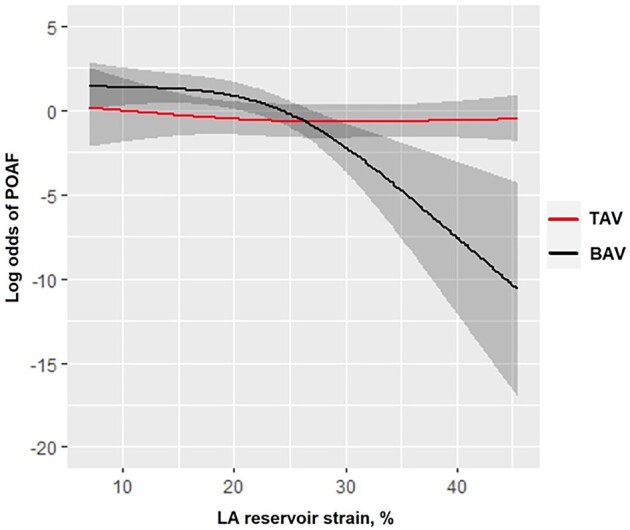
An interaction between aortic valve morphology and left atrial strain on post-operative atrial fibrillation. The association between left atrial strain and post-operative atrial fibrillation is displayed for bicuspid and tricuspid aortic valve patients.

Results from the multivariable logistic regression analysis revealed that pre-operative LA reservoir strain was independently associated with POAF after adjusting for age, gender, hypertension, diabetes, aortic valve morphology, pre-operative β-blocker treatment, pre-operative LVEF, pre-operative LA volume index, peak post-operative CRP, peak post-operative CK-MB, and post-operative inotropic support (*Table 4*). There was a significant interaction between LA reservoir strain and aortic valve morphology (BAV or TAV) on POAF probability (*P*_interaction_ = 0.002), indicating that the association between LA reservoir strain and POAF was more pronounced in BAV patients compared with TAV patients (*Figure 2*). The POAF incidence differed significantly between BAV and TAV patients depending on LA reservoir strain. For instance, the POAF incidence for BAV patients in the highest LA reservoir strain quartile (>28.0%) was 3%, while BAV patients in the lowest quartile (<15.8%) had a POAF incidence of 69.7%. The corresponding POAF incidence for TAV patients in the highest (>34.8%) and lowest LA reservoir strain (<21.9%) quartiles were 75.0 and 50.0%, respectively (see Supplementary material online, *Table S4*).

**Table 4:** Univariable and multivariable logistic regression analyses to assess the association between relevant variables and post-operative atrial fibrillation

	Univariable			Multivariable		
	Odds ratio	95% CI	*P-*value	Odds ratio	95% CI	*P-*value
Age, per 1 year increase	1.084	1.047–1.122	<0.001	1.064	1.023–1.106	0.002
Male gender	1.364	0.797–2.335	0.257	1.628	0.852–3.113	0.140
Hypertension	1.571	0.901–2.741	0.111	1.667	0.790–3.518	0.180
Diabetes mellitus	1.449	0.651–3.227	0.363	1.566	0.629–3.901	0.335
Aortic valve morphology (BAV as reference)	1.685	0.985–2.884	0.057	1.966	0.933–4.143	0.075
Pre-operative treatment with β-blocker	1.462	0.858–2.491	0.163	1.206	0.605–2.405	0.595
C-reactive protein, per 1 mg/mL increase	1.006	1.001–1.010	0.008	1.006	1.001–1.011	0.030
CK-MB, per 1 µg/L increase	1.026	1.003–1.050	0.026	1.025	0.999–1.052	0.061
Left ventricular ejection fraction, per 1%-point decrease	1.010	0.982–1.038	0.481	1.010	0.971–1.047	0.663
Left atrial volume index, per 1 mL/m^2^ increase	1.011	0.984–1.039	0.420	0.997	0.962–1.033	0.854
Left atrial reservoir strain, per 1%-point decrease	1.059	1.030–1.089	<0.001	1.064	1.032–1.095	<0.001
Post-operative inotropic support	0.988	0.552–1.770	0.968	0.619	0.304–1.263	0.188

BAV, bicuspid aortic valve; CK-MB, creatine kinase muscle brain.

Both LA reservoir strain (<24.4%) and impaired GLS (>−16%) were independently associated with POAF after adjusting for LA volume index >34 mL/m^2^, LVEF <50%, tricuspid regurgitation maximal velocity >2.8 m/s, and *E*/*e*′ >14 (*Table 5*). Left atrial reservoir strain <24.4% was also associated with persisting POAF, defined as AF at hospital discharge resistant to treatment with amiodarone and/or electrical cardioversion (*Table 6*).

**Table 5:** The incremental value of left atrial reservoir strain over standard echocardiographic parameters

Parameter	Odds ratio	95% confidence interval	*P-*value
LA reservoir strain <24.4%	6.48	2.62–16.04	<0.001
LA volume index >34 mL/m^2^	0.85	0.36–2.03	0.717
LVEF <50%	2.51	0.74–8.47	0.138
Global longitudinal strain >−16%	4.52	1.92–10.67	<0.001
TR Vmax >2.8 m/s	1.15	0.46–2.91	0.767
E/e′ > 14	1.25	0.55–2.85	0.598

LA, left atrium; LVEF, left ventricular ejection fraction, TR Vmax, tricuspid regurgitation maximal velocity.

**Table 6:** Multivariable logistic regression illustrating the risk for persisting post-operative atrial fibrillation, defined as persisting post-operative atrial fibrillation at discharge

Parameter	Odds ratio	95% confidence interval	*P-*value
LA reservoir strain <24.4%	11.93	1.39–102.10	0.024
LA volume index >34 mL/m^2^	1.86	0.36–9.74	0.460
LVEF <50%	1.81	0.50–7.05	0.349
Global longitudinal strain >−16%	0.96	0.27–3.42	0.954
TR Vmax >2.8 m/s	0.72	0.21–2.46	0.602
E/e′ > 14	1.05	0.34–3.28	0.934

LA, left atrium; LVEF, left ventricular ejection fraction, TR Vmax, tricuspid regurgitation maximal velocity.

### Post-operative TIA/stroke

**Figure 3:**
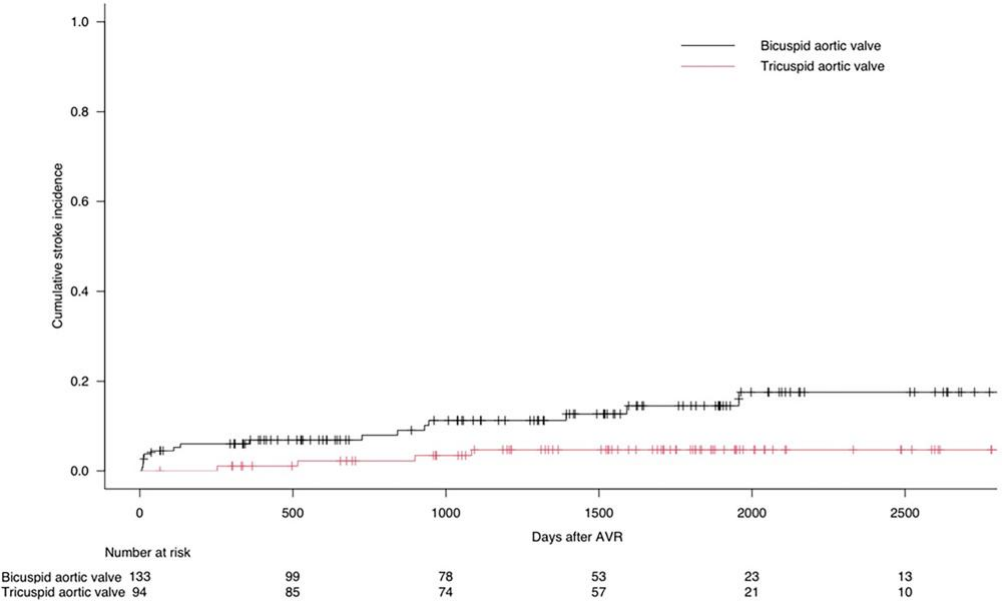
The cumulative TIA/stroke incidence for bicuspid aortic valve and tricuspid aortic valve patients after surgical aortic valve replacement, accounting for death as a competing risk. The cumulative TIA/stroke incidence during follow-up was significantly higher in bicuspid aortic valve patients (19.1 vs. 5.8% at 5 years, Fine–Gray *P* = 0.022).

Overall, there were 21 TIA/stroke events during follow-up, of which 16 occurred in BAV patients and 5 occurred in TAV patients (Fine–Gray *P* = 0.022). The cumulative TIA/stroke incidence for BAV and TAV patients is illustrated in *Figure 3*. No patient experienced a fatal stroke. There were two BAV patients who experienced disabling ischaemic strokes, all occurring late (>1 year) after index hospitalisation. Fourteen BAV patients had non-disabling stroke (*n* = 4) or TIA (*n* = 10). Two patients (one BAV and one TAV) also had a disabling haemorrhagic stroke, but these were not classified as stroke events in the present study. In an unadjusted, univariable Cox proportional hazards regression analysis, there was a significant association between left atrial volume index (LAVi) and TIA/stroke (HR = 1.044, 95% CI 1.013–1.077, *P* = 0.006 per 1 mL/m^2^ increase), but not for LA reservoir strain (HR = 1.030, 95% CI 0.987–1.074, *P* = 0.178 per 1% decrease).

## Discussion

The main finding of the present study was that pre-operative LA reservoir strain showed a significant interaction with aortic valve morphology with regard to POAF incidence after SAVR. Our results indicate that pre-operative LA function plays a major role in POAF development in BAV patients, while POAF development in TAV patients may be more multifactorial. Furthermore, the post-operative cumulative TIA/stroke incidence was significantly higher in BAV patients.

This longitudinal prospective study is the first to investigate the incidence of POAF in BAV and TAV AS patients separately. The overall POAF incidence (50.2%) was comparable with previous reports studying SAVR cohorts.^28,29^ Surprisingly, no significant difference in POAF incidence was observed between BAV and TAV patients, in spite of the significantly older TAV cohort. Furthermore, traditional cardiovascular risk factors such as hypertension, diabetes mellitus, hypercholesterolaemia, and obesity, as well as advanced age, all known to predispose to POAF,^1,30^ were more prevalent in TAV patients. Left atrial reservoir strain, on the other hand, was lower in BAV patients. Left atrial reservoir strain is known to be inversely correlated with LA fibrosis,^31^ and several studies suggest that impaired LA reservoir function is associated with POAF in patients with severe AS after aortic valve replacement, independently of LA enlargement.^14,15,22,32^ These results are in line with the findings of the present study (see Supplementary material online, *Table S5*). To date, this is the largest study investigating the association between LA reservoir strain and POAF after SAVR. We are also the first to investigate BAV and TAV patients separately, which is of great importance due to the vastly different pre-operative risk profiles.

We have recently demonstrated that BAV patients with isolated severe AS, without the influence of coronary artery disease, have lower LVEF and more prevalent diastolic dysfunction prior to SAVR compared with TAV counterparts.^21^ These findings, together with the chronicity of the BAV disease, might be the main cause behind the significant LA reservoir strain impairment observed in the BAV AS patients, where the atrial structural and electrical remodelling such as LA fibrosis constitutes a pre-existing vulnerable substrate for POAF. This might explain why the resolution of factors related to the surgical trauma was not as effective in converting POAF in BAV AS patients, where almost 30% were discharged with AF. In contrast, successful conversion to sinus rhythm was more common in TAV AS patients, which implies that POAF in TAV patients was more associated with transient post-operative alterations such as myocardial ischaemia, inflammation, and fluid overload. Even though peri-operative myocardial ischaemia, post-operative inflammation, and fluid overload were associated with POAF in the present study, there was no interaction with aortic valve morphology, suggesting that these triggers contribute to POAF in both BAV and TAV patients. Instead, there was a significant interaction between LA reservoir strain and aortic valve morphology on POAF probability, suggesting that LA reservoir function plays a major role in the development of POAF and the maintenance of AF in BAV patients. Likewise, POAF was rarely observed in BAV patients with preserved LA function. The close association between LA reservoir strain and POAF in BAV AS patients has some important implications. It could enable the identification of individuals who would benefit from prophylactic treatment, which is appealing as both β-blockers and amiodarone have some adverse side effects. More importantly, early identification of deteriorating LA reservoir strain is important, as early intervention might reduce the risk of POAF and thus its associated long-term complications.

Another interesting finding was the higher post-operative cumulative TIA/stroke incidence observed in BAV AS patients. This was also unexpected because BAV AS patients generally were healthier compared with TAV AS patients. However, since impaired LA reservoir strain is associated with stroke in both patients with sinus rhythm^33,34^ and AF,^35^ this should also be considered a possible explanation behind this difference. We also excluded patients with coronary artery disease, which often coexists with general atherosclerotic burden. Since coronary artery disease is typically more prevalent in TAV patients, the observed TIA/stroke incidence in the TAV cohort of the present study might not be representative of TAV AS patients in general. In an unadjusted Cox proportional hazards regression analysis, there was an association between LAVi and TIA/stroke. We hypothesize that patients with more dilated LA in the pre-operative setting are more prone to future AF events with resulting increased stroke risk, although this remains speculative. Due to the limited number of TIA/stroke events, we did not conduct a formal interaction analysis to assess whether LA dilatation affected the risk of TIA/stroke differently in BAV and TAV patients.

### Clinical implications and future directions

Post-operative atrial fibrillation remains a persistent complication despite advancements in prophylactic therapy. The results of the present study indicate that pre-operative LA function is associated with POAF in BAV AS patients, while the cause of POAF seems to be more multifactorial in TAV AS patients. It is possible that earlier surgical intervention to halt deteriorating LA function might reduce the risk of POAF in BAV AS patients, while modification of cardiovascular risk factors such as hypertension, diabetes, and obesity might reduce the risk of POAF in TAV patients. Furthermore, identifying the aetiology behind aortic valve degeneration in BAV and TAV AS could help identify therapies to slow the disease progression and limit AS-related complications, including POAF.^36^ We also found that the cumulative ischaemic TIA/stroke incidence during follow-up was higher in BAV AS patients compared with TAV AS patients, despite being younger, having a higher frequency of mechanical prostheses with warfarin treatment as well as having less comorbidities. The underlying causes need to be further elucidated.

### Limitations

The findings of the present study should be viewed in the context of some limitations. This was a single-centre study, and therefore, the results might not be generalised to other populations. Despite adjusting for relevant confounders, there were some risks of residual confounding due to the observational design of the study. We did not analyse post-operative LA reservoir strain. Recovery of LA reservoir strain in the immediate post-operative period could potentially be used to discriminate for which patients the reduced LA reservoir strain is mainly caused by the increased afterload induced by the AS, rather than the elevated end-diastolic pressure caused by adverse LV remodelling. This should be investigated in future studies.

## Conclusions

The POAF incidence after SAVR did not differ between BAV AS and TAV AS patients. Pre-operative LA function was associated with POAF after SAVR in BAV AS patients, while POAF in TAV AS patients likely depended on transient post-operative alterations and traditional cardiovascular risk factors. Routine quantification and surveillance of LA reservoir strain could be important in BAV AS patients to determine optimal timing for SAVR and reduce POAF incidence.

## Lead author biography


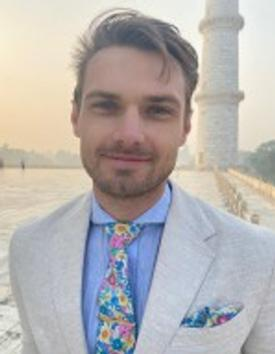


Johan O. Wedin, MD, is a resident physician and PhD candidate in Cardiothoracic Surgery at the Department of Cardiothoracic Surgery and Anesthesiology, Uppsala University Hospital, Uppsala, Sweden. He also has a bachelor’s degree in Biomedical Laboratory Science and Clinical Physiology and has previously worked as a cardiac sonographer. His research focuses on characterizing echocardiographic, surgical, and bioinformatic differences between patients with bicuspid and tricuspid aortic stenosis.

## Supplementary Material

oeae020_Supplementary_Data

## Data Availability

The analysed dataset, underlying this manuscript, will be shared on reasonable request to the corresponding author.
