# Supplementary material for: Left atrial dysfunction in bicuspid aortic valve patients with severe aortic stenosis is associated with post-operative atrial fibrillation following aortic valve replacement
Source: Eur Heart J Open. 2024 Mar 28;4(2):oeae020. doi: 10.1093/ehjopen/oeae020 (PMC11000824; doi:10.1093/ehjopen/oeae020)
Supplement: oeae020_Supplementary_Data [file oeae020_supplementary_data.docx]

# SUPPLEMENTARY MATERIAL

Left atrial dysfunction in bicuspid aortic valve patients with severe aortic stenosis is associated with postoperative atrial fibrillation following aortic valve replacement

# Johan O. Wedin, Sergey Rodin, Frank A. Flachskampf, Oscar E. Simonson, Johan Pallin, Jonathan Hörsne Malmborg, Stefan K. James, Elisabeth Ståhle, Karl-Henrik Grinnemo

# **EXPANDED METHODS**

## **Echocardiographic imaging**

All patients underwent a comprehensive preoperative two-dimensional transthoracic echocardiogram according to current recommendations for evaluation of left ventricular (LV) size and systolic function (1), LV global longitudinal strain (2) as well as diastolic function (3).

***Chamber quantification and left ventricular systolic function***
Left ventricular (LV) linear dimensions were obtained from a standard parasternal long-axis view in systole (LV end-systolic diameter) and diastole (interventricular septal thickness, posterior wall thickness and LV end-diastolic diameter). LV mass was calculated using the Devereux formula and indexed to body surface area (BSA). LV end-diastolic volume (LVEDV) and LV end-systolic volume (LVESV) were obtained from the biplane disk summation by tracing of the endocardial border in the apical 4- and 2-chamber views. The LV volumes were indexed to BSA. The global LV systolic function was estimated with the LV ejection fraction (LVEF), which was obtained using the Simpson’s biplane method of disks. Biplane left atrial (LA) maximal volume was measured in end-systole, avoiding foreshortening of the atrium, in the apical 4- and 2-chamber views using the Simpson’s method. The pulmonary veins and the left atrial appendage were excluded from the tracing. The biplane LA volume was indexed to BSA.

### ***Left ventricular global longitudinal strain***

Left ventricular global longitudinal strain (GLS) was measured from apical 2-, 4- and 3-chamber views. The endocardial border was traced in the end-systolic frame, while the end-diastolic tracing was provided automatically by the software, allowing for manual correction if necessary. GLS calculation was not performed if the frame rate was <40 or if the tracking was suboptimal in more than two segments.

***Left ventricular diastolic function***
The diastolic function was classified as normal, indeterminate or impaired. The diagnosis of diastolic dysfunction was based on the combination of 4 parameters; the E to e’ ratio (E/e’), septal and lateral e’ velocities, maximal velocity of the tricuspid regurgitant signal (TR Vmax) and LA volume indexed to BSA (LAVi) with the following cut off values:

- E/e’_average_ >14 or E/e’_septal_ >15 or E/e’_lateral_  >13
- Septal e’ velocity <7 cm/s or lateral e’ velocity <10 cm/s
- TR Vmax >2.8 m/s
- LAVi >34 mL/m^2^

Diastolic function was normal if 1/4 criteria were positive, indeterminate if 2/4 criteria were positive, and impaired if 3-4/4 criteria were positive.

**Surgical intervention and per- and postoperative anesthesiologic care**
Surgical aortic valve replacement was performed through a full median sternotomy with the use of standard cardiopulmonary bypass. The choice of prosthesis was at the discretion of the surgeon, adhering to the current recommendations (4), with the patient´s preference in mind. Temporary pacemaker electrodes were placed on the right ventricle to prevent postoperative bradycardia, and none of the patients were treated with bi-atrial pacing. Performing a posterior left pericardiotomy for PAOF prevention is not routine at out department, and this was not conducted in the present study.

Our department routinely use norepinephrine as primary inotropic agent to counteract hypotension and resulting hypoperfusion in the per- and postoperative period. Inotropic agents, including norepinephrine, are associated with POAF through the sympathetic drive. We therefore make our best effort to reduce the use of norepinephrine. In this study, the use of inotropic support was defined as the need for norepinephrine >24 h.

## **POAF prevention**

Our department do not use a specific protocol for initiation of pharmacological prophylaxis for POAF prevention in drug-naïve patients. Our preventive strategies include optimizing fluid balance, correct electrolyte imbalances and minimizing the use of inotropic agents. For patients on preoperative β-blocker therapy, treatment was continued immediately postoperative unless contraindicated due to bradycardia.

## **Postoperative laboratory measurements and investigations**

## C-reactive protein (CRP) and creatine kinase muscle-brain (CK-MB) levels were measured at least the first three postoperative days and longer if necessary, and peak values were recorded. Body weight was recorded prior to breakfast every day to monitor postoperative fluid balance. All patients received standard postoperative treatment with 40 mg furosemide and 25 mg spironolactone from the first postoperative day. These doses were adjusted daily according to changes in body weight. A conventional chest x-ray was performed on the second postoperative day to rule out significant pneumothorax or pleural effusion. A TTE was performed on the third postoperative day to rule out significant pericardial effusion, paravalvular leaks and patient-prosthesis mismatch.

# **EXPANDED RESULTS**

**Table S1** summarizes difference between patients with and without POAF with regard to aortic valve morphology. BAV patients who developed POAF (*n*=64) were older (68.1 years vs. 62.7 years, *P*<0.001), had lower LA reservoir strain (16.9% vs. 27.1%, *P*<0.001), were less likely to be tobacco smokers (28.1% vs. 49.3%, *P*=0.013), had higher postoperative CRP levels (219 mg/L vs. 193 mg/L, *P*=0.027) and had a significantly higher postoperative weight gain (4.3 kg vs. 3.0 kg, *P*<0.001) compared to BAV patients who remained in sinus rhythm (*n*=69). TAV patients who developed POAF (*n*=50) were older (73.5 years vs. 68.6 years, *P*<0.001), had higher postoperative CRP levels (207 mg/L vs. 175 mg/L, *P*=0.005), higher CK-MB levels (28 µg/L vs. 18 µg/L, *P*=0.031), and a higher frequency of significant postoperative pericardial effusion (14.0% vs. 0.0%, *P*=0.014) than TAV patients who remained in sinus rhythm (*n*=44).

# **ADDITIONAL REFERENCES**

1. Lang RM, Badano LP, Mor-Avi V, Afilalo J, Armstrong A, Ernande L, et al. Recommendations for cardiac chamber quantification by echocardiography in adults: an update from the American Society of Echocardiography and the European Association of Cardiovascular Imaging. J Am Soc Echocardiogr Off Publ Am Soc Echocardiogr. 2015 Jan;28(1):1-39.e14.

2. Voigt JU, Pedrizzetti G, Lysyansky P, Marwick TH, Houle H, Baumann R, et al. Definitions for a common standard for 2D speckle tracking echocardiography: consensus document of the EACVI/ASE/Industry Task Force to standardize deformation imaging. J Am Soc Echocardiogr Off Publ Am Soc Echocardiogr. 2015 Feb;28(2):183–93.

3. Nagueh SF, Smiseth OA, Appleton CP, Byrd BF, Dokainish H, Edvardsen T, et al. Recommendations for the Evaluation of Left Ventricular Diastolic Function by Echocardiography: An Update from the American Society of Echocardiography and the European Association of Cardiovascular Imaging. J Am Soc Echocardiogr Off Publ Am Soc Echocardiogr. 2016 Apr;29(4):277–314.

4. Vahanian A, Beyersdorf F, Praz F, Milojevic M, Baldus S, Bauersachs J, et al. 2021 ESC/EACTS Guidelines for the management of valvular heart disease. Eur Heart J. 2022 Feb 12;43(7):561–632.

**
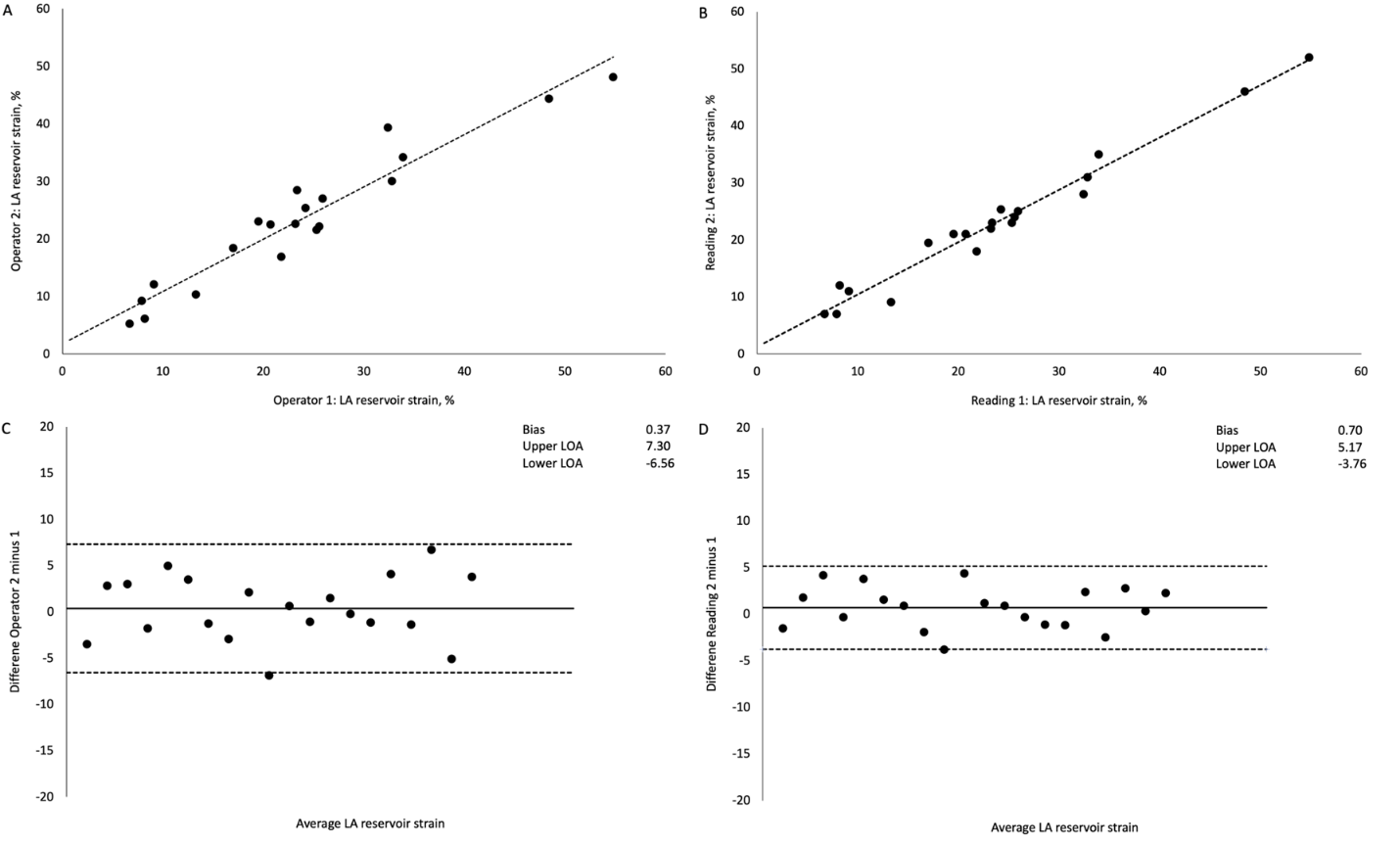
**

**Figure S1.** Pearson *r* and Bland-Altman analysis were performed to investigate correlation and inter- and intra-observer agreement for repeated measures of LA reservoir strain. A) The inter-observer and B) intra-observer variability for LA reservoir strain were very strong with Pearson *r* >0.90. The C) inter-observer and D) intra-observer agreement were very strong (intra-class correlation coefficient >0.90) with narrow limits of agreement and no evidence of bias.

**
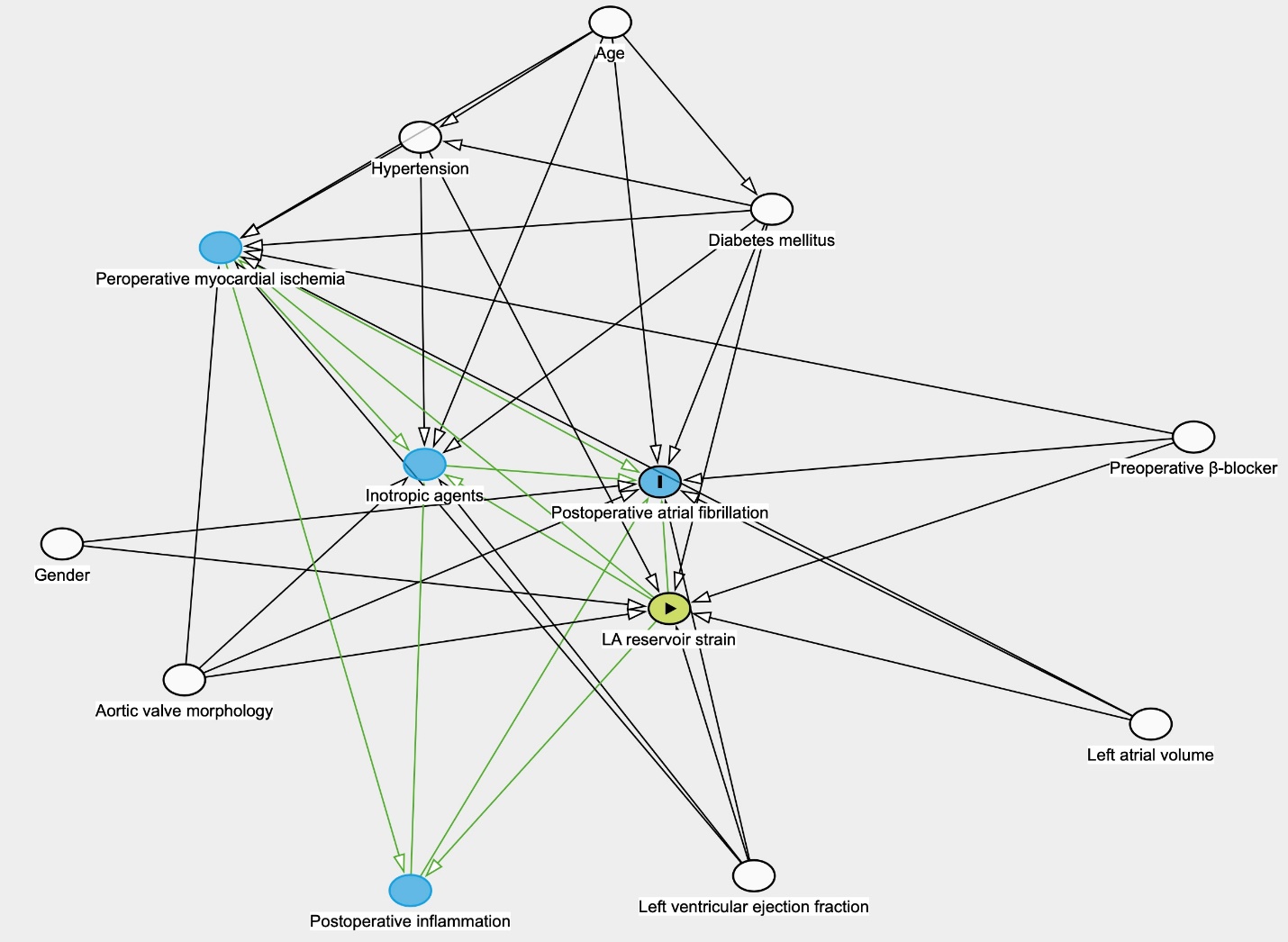
**

**Figure S2.** Directed acyclic graph was constructed to select relevant covariates to adjust for in the multivariable regression analysis.

**Table S1. Reproducibility for LA reservoir strain.**

|  | ICC coefficient | 95% CI | *P* value |
| --- | --- | --- | --- |
| Intra-observer ICC | 0.984 | 0.961-0.994 | <0.001 |
| Inter-observer ICC | 0.978 | 0.946-0.991 | <0.001 |

Abbreviations: CI, confidence interval; ICC, intra-class correlation.

**Table S2. Characteristics of BAV and TAV patients with POAF.**

|  | **Bicuspid aortic valve**  **(*n* = 64)** | **Tricuspid aortic valve**  **(*n* = 50)** | ***P* value** |
| --- | --- | --- | --- |
| Age, years | 68.1 (7.9) | 73.5 (5.3) | **<0.001** |
| Male gender, *n* (%) | 45 (70.3) | 31 (62.0) | 0.350 |
| Body mass index, kg/m^2^ | 27.0 (4.4) | 28.1 (3.6) | 0.146 |
| CHA2DS2VASC, score | 2.81 (1.41) | 3.66 (1.45) | **0.002** |
| LVEF, % | 53 (11) | 60 (6) | **<0.001** |
| GLS, % | -14.3 (3.8) | -18.1 (5.9) | **<0.001** |
| LA volume index, mL/m^2^ | 41 (8.1) | 37 (10.4) | **0.026** |
| LA reservoir strain, % | 16.9 (6.9) | 27.1 (9.5) | **<0.001** |
| Weight increase from baseline, kg | 4.3 (2.2) | 3.9 (2.1) | 0.183 |
| Significant pericardial effusion, *n* (%) | 9 (14.1) | 7 (14.0) | 0.992 |
| Significant pneumothorax, *n* (%) | 2 (3.1) | 2 (4.0) | 0.801 |
| Maximal postoperative C-reactive protein level, mg/L | 219 (67) | 206 (59) | 0.144 |
| Maximal postoperative CK-MB level, µg/L | 38 (97) | 29 (28) | 0.368 |
| ICU, days | 2.84 (3.33) | 2.00 (1.69) | 0.082 |
| Inotropic support, *n* (%) | 21 (32.8) | 8 (16.0) | **0.041** |
| Nitroglycerin infusion, *n* (%) | 11 (17.2) | 12 (24.0) | 0.368 |
| Time to POAF onset, days | 2.72 (2.28) | 2.49 (1.08) | 0.441 |
| Treatment with amiodarone, *n* (%) | 48 (75.0) | 41 (82.0) | 0.264 |
| Treatment with electric cardioversion, *n* (%) | 31 (48.4) | 17 (34.0) | 0.143 |
| Spontaneous conversion to sinus rhythm, *n* (%) | 7 (9.4) | 10 (20.0) | 0.095 |
| Persisting POAF at discharge, *n* (%) | 19 (29.7) | 4 (8.0) | **0.005** |

Abbreviations: POAF; postoperative atrial fibrillation; LVEF, left ventricular ejection fraction; GLS, global longitudinal strain; LA, left atrium; CK-MB, creatine kinase muscle-brain; ICU, intensive care unit.

**Table S3. Differences between patients with and without POAF with regard to aortic valve morphology.**

|  | **Bicuspid aortic valve w/o POAF**  **(*n* = 69)** | **Bicuspid aortic valve w/ POAF**  **(*n* = 64)** | ***P* value** | **Tricuspid aortic valve w/o POAF**  **(*n* = 44)** | **Tricuspid aortic valve w/ POAF**  **(*n* = 50)** | ***P* value** |
| --- | --- | --- | --- | --- | --- | --- |
| Age, years | 62.7 (9.5) | 68.1 (8.0) | **<0.001** | 68.6 (8.0) | 73.5 (5.3) | **<0.001** |
| Male gender, *n* (%) | 42 (60.9) | 45 (70.3) | 0.253 | 22 (50.0) | 31 (62.0) | 0.242 |
| Body mass index, kg/m^2^ | 27.1 (4.7) | 27.0 (4.4) | 0.914 | 28.5 (4.4) | 28.1 (3.6) | 0.645 |
| Hypertension, *n* (%) | 40 (58.0) | 37 (57.8) | 0.985 | 33 (75.0) | 42 (84.0) | 0.278 |
| Diabetes mellitus, *n* (%) | 4 (5.8) | 8 (12.5) | 0.231 | 11 (25.0) | 6 (12.0) | 0.102 |
| Chronic obstructive pulmonary disease, *n* (%) | 3 (4.4) | 5 (7.8) | 0.481 | 2 (4.5) | 8 (16.0) | 0.098 |
| Hypercholesterolemia, *n* (%) | 26 (37.7) | 19 (29.7) | 0.330 | 26 (59.1) | 26 (52.0) | 0.559 |
| CHA2DS2VASC, score | 2.35 (1.52) | 2.81 (1.41) | 0.075 | 3.11 (1.22) | 3.66 (1.45) | 0.053 |
| Smoking, *n* (%) | 34 (49.3) | 18 (28.1) | **0.013** | 19 (43.2) | 19 (38.0) | 0.609 |
| LVEF, % | 56 (11) | 53 (11) | 0.117 | 61 (7) | 60 (6) | 0.224 |
| GLS, % | -14.4 (4.2) | -14.3 (3.8) | 0.946 | -18.0 (4.4) | -18.0 (5.9) | 0.949 |
| LA volume index, mL/m^2^ | 40 (11.5) | 41 (8.0) | 0.602 | 35 (7.3) | 37 (10.4) | 0.219 |
| LA reservoir strain, % | 27.1 (11.5) | 16.9 (6.9) | **<0.001** | 29.3 (9.3) | 27.0 (9.5) | 0.260 |
| Weight increase from baseline, kg | 3.0 (1.8) | 4.3 (2.2) | **<0.001** | 3.1 (1.7) | 3.9 (2.1) | 0.054 |
| Significant pericardial effusion, *n* (%) | 3 (4.3) | 9 (14.1) | 0.069 | 0 (0.0) | 7 (14.0) | **0.014** |
| Significant pneumothorax, *n* (%) | 3 (4.4) | 2 (3.1) | 0.711 | 0 (0.0) | 2 (4.0) | 0.497 |
| Peak postoperative C-reactive protein level, mg/L | 193 (67) | 219 (67) | **0.027** | 175 (53) | 207 (57) | **0.005** |
| Peak postoperative CK-MB level, µg/L | 21 (9) | 38 (97) | 0.138 | 18 (9) | 28 (28) | **0.031** |
| ICU, days | 1.61 (1.20) | 2.84 (3.32) | **0.005** | 1.32 (1.03) | 2.00 (1.69) | **0.022** |
| Inotropic support, *n* (%) | 17 (24.6) | 21 (32.8) | 0.297 | 4 (9.0) | 8 (16.0) | 0.368 |
| Nitroglycerin infusion, *n* (%) | 8 (11.6) | 11 (17.2) | 0.357 | 15 (34.1) | 12 (24.0) | 0.281 |

Abbreviations: POAF; postoperative atrial fibrillation; LVEF, left ventricular ejection fraction; GLS, global longitudinal strain; LA, left atrium; CK-MB, creatine kinase muscle-brain; ICU, intensive care unit.

**Table S4. POAF incidence in BAV and TAV AS patients according to LA reservoir strain quartiles.**

|  | **Bicuspid aortic valve** | **Tricuspid aortic valve** |
| --- | --- | --- |
| Quartile 1 (BAV, <15.8%; TAV, <21.9) | 69.7% (23/33) | 75.0% (18/24) |
| Quartile 2 (BAV, 15.9-21.9%; TAV, 22.0-26.6%) | 67.6% (23/34) | 60.9% (14/23) |
| Quartile 3 (BAV, 22.0-27.9%; TAV, 26.7-34.7%) | 54.5% (18/33) | 62.5% (15/24) |
| Quartile 4 (BAV, >28%; TAV, >34.8%) | 3.0% (1/33) | 50% (12/24) |

Abbreviations: BAV, bicuspid aortic valve; TAV, tricuspid aortic valve.

**Table S5.**

| **Study** | **Design** | **Sample size** | **POAF incidence** | **Discrimination between BAV and TAV** | **Adjusted OR/HR** | **AUC** | **Cutoff** | **Sensitivity, specificity** |
| --- | --- | --- | --- | --- | --- | --- | --- | --- |
| Pernigo et al^14^ | Prospective | 60 | 43.3% (26/60) | No* | 0.75 (OR) | 0.87 | <23% | NA |
| Pessoa-Amorim et al^15^ | Prospective | 114 | 31.6% (36/114) | No^‡^ | 0.97 (HR) | NA | <18.7% | NA |
| Cameli et al^11^ | Prospective | 76 | 19.7% (15/76) | No | 0.91 (HR) | 0.89 | <16.9% | 86%, 91% |
| Imanischi et al^32^ | Retrospective | 27 | 55.6% (15/27) | No | 0.01 (HR) | 0.83 | 0.79 s^-1^ | 60%, 92% |
| Wedin et al | Prospective | 227 | 50.2% (114/227) | Yes | 1.06^§^ (OR) | BAV: 0.79  TAV: 0.57 | BAV: <24.4%  TAV: <25.2% | BAV: 91%, 65% TAV: 48%, 59% |

Abbreviations: AUC, area under curve; BAV, bicuspid aortic valve; HR, hazard ratio; NA, not available; OR, odds ratio; POAF, postoperative atrial fibrillation; TAV, tricuspid aortic valve.

* Only reported the frequency of BAV in POAF and non-POAF groups.

^‡^ BAV served as exclusion criteria.

^§^ We reported the OR per 1%-decrease in LA reservoir strain in contrast to the other studies which reported OR/HR per 1%-increase in LA reservoir strain (Imanschi et al., reported HR for LA reservoir strain rate).
